# Keystone Veterinary Medical Association

**Published:** 1897-06

**Authors:** W. L. Rhoads

**Affiliations:** Secretary


Secretary.

### KEYSTONE VETERINARY MEDICAL ASSOCIATION.

THE March meeting was called to order by President John R. Hart, on the evening of the 9th, with the following members of the profession present: Drs. W. W. Martin, H. D. Martein, J. D. Houldsworth, Clarence J. Marshall, Thomas B. Rayner, W. L. Rhoads, Charles Lintz, W. S. Kooker, W. H. Hoskins, John R. Hart, Charles T. Goentner, F. S. Allen, and a number of students from the Veterinary Department of the University of Pennsylvania. We also had the pleasure of having with us Professor Marks, of the Adirondack Valley, and Mr. S. C. Damond, of Massachusetts, and, as speaker of the evening, R. A. Pearson, Assistant Chief of the Dairy Division of the Department of Agriculture, Washington, D. C.

After the calling of the roll, reading and adopting of the minutes of the February meeting, the regular order of business was suspended that Mr. R. A. Pearson might read his paper on "Sanitary Milk." This was followed by a paper by Dr. Leonard Pearson, on "What Professor Bang's Work Teaches." [The papers have been printed in full in the April and May numbers of the JOURNAL.]

Dr. Pearson having been called to Harrisburg, his paper was read by the Secretary. After its reading, discussion was entered into upon both papers. After the discussion a hearty vote of thanks was extended to Dr. R. A. Pearson, when adjournment was in order.

The April meeting was called to order by Vice-President James B. Rayner, upon whom the presidential duties now rest, through the irreparable loss to the Association by the death of our late coworker, esteemed fellow-member, and beloved President, John R. Hart.

Though the attendance at this meeting was light in number, those who were there brought hearts full of sorrow. Individually realizing the loss to each of us was but a fraction of that sustained by the profession as a whole, and this could be but a unit of the loss to the bereaved family of our worthy colleague who, as a reward for his earnest, honest work here, was taken to a brighter, happier home. Though the majority of those present had few words to express their sorrow, this was due to their having realized the loss to the profession and themselves individually, mingled with the feeling that we collectively must be a long time doing what he might have done alone, knowing that it would take a long time for his most earnest friends and coworkers to close up the gap left by his moving onward and upward to a new life. Every one seemed impressed with his broad-minded, full-hearted, and conscientious life; a life which could well be held up as an example to every young man; a life which was not a theory, but a living proof of what could be done by an honest man. It has been said that an honest man was the noblest of God's handiwork, and surely if such exists or has existed, Dr. John R. Hart was of the number, and we cannot say more of him than that which we hear from every side, among all classes, and all walks of life —" He was an honest man." Drs. W. H. Hoskins, F. S. Allen, and W. S. Kooker were appointed as a committee to draft resolutions upon the death of Dr. John Hart.

Some voluntary contributions were received toward the floral emblem sent to the family of our late President as a mark of sympathy, sorrow, and respect by the K. V. M. A., and it was orderd by a vote of the Association that the balance be paid out of the Association funds.

The May meeting was called to order by Dr. Thomas B. Rayner, as Vice-President James B. Rayner was not present. The members of the profession in attendance were: Drs. Charles M. Cullen, H. P. Eves, H. D. Martein, W. H. Hoskins, Charles Lintz, John W. Adams, Thomas B. Rayner, and W. L. Rhoads. Dr. W. H. Hoskins, as chairman of the Committee on Resolutions, reported progress.

Dr. Hoskins then brought to the notice of the Association, under the head of new business, the disposition to ignore the civil service system in government offices and the re-establishment of the spoils system in our national civil service, and asked that the sentiment of the meeting might be conveyed to Secretary of Agriculture Wilson and to Senator Pritchett as well as all others likely to be interested.

He also spoke of the bill now pending for the purpose of establishing a number of inspectors along the border line of the State and the taxation of all carcasses coming in at $2.00 per head.

The applications of Dr. John W. Adams and H. D. Martein for membership were now read and referred to the Board of Censors.

Dr. John W. Adams was now introduced and addressed the Association on meat-inspection as it applies to large cities. After speaking of the various diseases met with, making beef, veal, mutton, and pork unfit for food, he then went on to tell of the inspection of Philadelphia's meat-supply, which, despite the best efforts of the inspectors, is at best a farce, when we appreciate the fact that Philadelphia has over one hundred and fifty slaughter-houses, and but four inspectors, the chief of whom is a layman, and his assistant a deputy with no special qualifications. The other members of the inspection force are two veterinarians, who are finally called to pass upon carcasses if questioned. This inadequate force is augmented by a consulting veterinarian, whose services are seldom called upon.

The great number of slaughter-houses makes it impossible for the inspectors to complete their rounds in a week, yet every day slaughter goes on in these places. Better inspection is needed, central abattoirs are a necessity. District slaughter-houses will be the only means of bettering the present conditions. He then spoke of the meat-inspection of the large cities of Germany. In the abattoirs of Berlin animals overheated or footsore are not allowed to be slaughtered. The deputy goes first, then the inspector comes, and he examines liver, lungs, and parts of carcass. If right, it is stamped upon the forearm, hip, region of tibia, etc.; if meat is found without this stamp, a question is immediately raised and a fine imposed. The inspection of pork for trichina is especially thorough, four sections being taken and subjected to microscopical test. Calves are watched: intestines and mesenteric glands for tuberculosis; lungs for verminous bronchitis; if lean and unthrifty, are condemned on general principles. You cannot buy bruised or unhealthy meat of any kind. The horse meat to be sold must go under the same rigid examination, and cannot be sold from the same store as other meats. He said by placing meat in cold-storage the odor was kept down, and all meat coming from these places should be closely watched. He has seen here slaughter-houses in full operation with open doors, with the next houses infected with scarlatina, measles, diphtheria, etc., for the germs of which we have no better media than fresh raw meat. Thus, every section into which the meat went was liable to a scourge of these diseases. The general public are entitled to better protection, and the best way to give it is first to awaken them to the true state of affairs through the columns of the press, and, as the medical profession, as a rule, seem indifferent to the present state of affairs as regards the meat- and milk-supply of the community from which they not only gain a livelihood, but many of them a competency, it becomes the duty of the veterinarian as a humanitarian to do all in his power to improve the present existing conditions. During the general discussion the present deplorable state of affairs was more clearly shown. Much of the meat now exposed for sale has the diseased parts trimmed off. The Chicago meat, as a rule, is better than home-dressed meat, as the inspection is more thorough. While the taxpayer and meat consumer feels secure, thinking the city fathers are protecting his health by thorough meat- and milk-inspection, this responsibility is really thrown upon the butcher, vendor, or producer.

Professor Marks said that in his experience he found the veterinarians do not stand up for their rights; they are not aggressive enough, but will stand back and see positions which they alone are qualified to fill held by medical men, laymen, or quacks who have a political pull.

Dr. Cullen felt the best way to remedy the present condition was by the coworking of the veterinarians, the employment of legal talent, and the use of the press. In answer to some questions regarding sheep, it was said that the lungs were used for making soup in Germany, and in the inspection these were closely watched for verminous bronchitis, also that gid or turnsick was due to a cerebral cyst caused by tænia cœnurus. This disease is directly transmissible to man, thus making the meat of these animals unfit for food.

The nodular disease of sheep was really intestinal tumors caused by invasion of a parasite, and in some sections of the South made sheep husbandry unprofitable. These tumors resemble tubercular nodules, and may be mistaken for them.

Dr. Hoskins now reported a case of a collie dog having frequent micturition, each time ending with a few drops of blood; this was finally cured by charging his drinking-water with bicarbonate of sodium, and dosing with sanmetto.

Dr. H. D. Martein exhibited some fine specimens of calculi taken from horses upon which he had held post-mortems at the Zoo.

After the extension of a hearty vote of thanks to Dr. Adams for his kindness as speaker of the evening, the Association adjourned, to meet June 15, 1897.

W. L. Rhoads,
Secretary.

